# Transitioning From In-Person to Remote Clinical Research on Depression and Traumatic Brain Injury During the COVID-19 Pandemic: Study Modifications and Preliminary Feasibility From a Randomized Controlled Pilot Study

**DOI:** 10.2196/28734

**Published:** 2021-12-01

**Authors:** Lauren B Fisher, Sylvie Tuchman, Andrew J Curreri, Maggie Markgraf, Maren B Nyer, Paolo Cassano, Grant L Iverson, Maurizio Fava, Ross D Zafonte, Paola Pedrelli

**Affiliations:** 1 Department of Psychiatry Massachusetts General Hospital Boston, MA United States; 2 Department of Psychiatry Harvard Medical School Boston, MA United States; 3 Department of Psychological & Brain Sciences Boston University Boston, MA United States; 4 Department of Physical Medicine and Rehabilitation Harvard Medical School Boston, MA United States; 5 Department of Physical Medicine and Rehabilitation Spaulding Rehabilitation Hospital Boston, MA United States; 6 Red Sox Foundation and Massachusetts General Hospital Home Base Program Boston, MA United States; 7 Department of Physical Medicine and Rehabilitation Massachusetts General Hospital and Brigham and Women’s Hospital Boston, MA United States

**Keywords:** COVID-19, telemental health, clinical trial, traumatic brain injury, depression, cognitive behavioral therapy

## Abstract

**Background:**

Telehealth has provided many researchers, especially those conducting psychosocial research, with the tools necessary to transition from in-person to remote clinical trials during the COVID-19 pandemic. A growing body of research supports the effectiveness of telemental health for a variety of psychiatric conditions, but few studies have examined telemental health for individuals with comorbid medical diagnoses. Furthermore, little is known about the remote implementation of clinical trials examining telemental health interventions.

**Objective:**

This paper outlines the procedural modifications used to facilitate conversion of an in-person randomized controlled trial of cognitive behavioral therapy (CBT) for depression in individuals with traumatic brain injury (TBI; CBT-TBI) to a telemental health study administered remotely.

**Methods:**

Given the nature of remote implementation and specific challenges experienced by individuals with TBI, considerations related to treatment delivery, remote consent, data management, neuropsychological assessment, safety monitoring, and delivery of supportive material have been discussed. Feasibility, acceptability, and safety were evaluated by examining attendance and participant responses on self-report measures of treatment satisfaction and suicidal behavior.

**Results:**

High rates of treatment attendance, assessment completion, study retention, and satisfaction with the intervention and modality were reported by participants who completed at least one telemental health CBT-TBI session.

**Conclusions:**

Study modifications are necessary when conducting a study remotely, and special attention should be paid to comorbidities and population-specific challenges (eg, cognitive impairment). Preliminary data support the feasibility, acceptability, and safety of remotely conducting a randomized controlled trial of CBT-TBI.

**Trial Registration:**

ClinicalTrials.gov NCT03307070; https://clinicaltrials.gov/ct2/show/NCT03307070

## Introduction

The COVID-19 pandemic has had significant impacts on the conduct of clinical research. As a result of stay-at-home orders, most in-person clinical trials for nonlifesaving interventions were suddenly halted, leaving many active research participants and teams in limbo. Many researchers were forced to choose between pausing their research projects, with a threat to scientific productivity, and modifying procedures to implement remote approaches [1].

Psychosocial research (eg, investigation of psychological treatments) is well positioned to be conducted remotely. Telemental health, the application of telecommunications to provide mental health services from a distance, has grown exponentially during the COVID-19 pandemic [2] and includes the use of a wide range of technologies to deliver synchronous (eg, live videoconferencing or telephone calls) and asynchronous interventions (eg, web-based interventions completed without a clinician present) [3]. This paper primarily focuses on the use of synchronous exchanges with telephone and videoconferencing to facilitate the remote implementation of assessment and psychotherapy in the context of a clinical trial for depression that was conducted in-person prior to the pandemic. The transition to remote study implementation was supported by a growing body of research demonstrating the effectiveness of telemental health services across many populations (eg, adults, children, and older adults) and for a range of psychiatric conditions [4], including depression [5], anxiety [6], and posttraumatic stress disorder [7]. In fact, real-time telemental health (ie, videoconferencing or telephone) is as effective as face-to-face treatment in reducing depressive symptoms [5,8]. Furthermore, treatment satisfaction and therapeutic alliance are similar among patients engaged in telemental health (videoconferencing and telephone-based interventions) and in-person treatment [9]. Effective implementation of protocols for remotely assessing and managing suicide risk further supports the feasibility of conducting clinical trials that examine telemental health interventions for individuals with depression [10].

Despite the significant promise of telemental health for many individuals with depression, individuals with various comorbid medical diagnoses may experience distinct challenges that serve as barriers to effectively utilizing telemental health interventions and participating in clinical research implemented remotely. In our work with individuals who have sustained traumatic brain injury (TBI), the impacts of TBI sequelae, including cognitive difficulties (eg, impaired focus and attention, and executive dysfunction) and sensitivity to light/screens, present unique challenges to participation in telemental health. Nevertheless, preliminary evidence suggests that individuals with major depressive disorder (MDD) and complicated mild to severe TBI experience similar reductions in depressive symptoms after 16 weeks of in-person and telephone-delivered cognitive behavioral therapy (CBT), and report similarly high rates of treatment satisfaction and strong therapeutic alliance [11]. Although we have been unable to identify studies examining the use of videoconferencing for the delivery of individual psychotherapy for adults with depression and TBI, there is support for the feasibility of using videoconferencing for group CBT to improve emotion regulation after TBI [12] and problem solving–based telemental health for improving behavior and family functioning in children with TBI [13]. Furthermore, support for the feasibility of video-based telerehabilitation in adults with TBI [14] suggests that telemental health interventions adapted for this population may also be feasible.

Given the mounting evidence for telemental health research as a feasible alternative to face-to-face participation in clinical trials and the public health restrictions during the COVID-19 pandemic, in March 2020, we transitioned our National Institutes of Health (NIH)-funded, in-person randomized controlled trial (RCT) of CBT for depression in individuals with TBI (CBT-TBI) to a remotely delivered telemental health study. In this paper, we discuss the study modifications that were implemented to maximize the feasibility, acceptability, and safety of remote study participation for individuals with depression and TBI. Preliminary evidence of feasibility, acceptability, and safety is examined among individuals who began the study in-person and transitioned to remote procedures, as well as participants who completed all procedures remotely.

## Methods

### Participants

Study participants were enrolled in an ongoing randomized waitlist-controlled trial (target N=40) piloting a 12-week individual CBT for depression that was adapted for individuals with TBI. The aims of the parent trial were to evaluate the feasibility and acceptability of the intervention (primary), as well as the potential efficacy in reducing depressive severity (secondary). As of August 27, 2021, the ongoing RCT enrolled (consented) a total of 33 participants, of which 18 participants were enrolled in-person and 15 participants were enrolled remotely. The clinical trial began in-person recruitment in April 2019 and transitioned to remote procedures on March 16, 2020, which remains ongoing at the time of writing. Of the 33 enrolled participants, 8 completed all study visits in-person, 6 completed a combination of in-person and telemental health sessions, 2 were enrolled in-person but completed all CBT-TBI sessions remotely, 7 completed all study visits remotely, 3 were deemed ineligible at the screening session (1 in-person and 2 remote), 2 discontinued (during remote CBT-TBI), 1 was lost to follow-up, and 3 determined they did not have time to participate (immediately after remote enrollment). One participant remains active at the time of writing. The feasibility and safety analyses presented below include participants who completed one or more CBT-TBI sessions remotely. Acceptability data include participants who completed one or more CBT-TBI sessions remotely and completed the study (defined as attending all 12 intervention sessions; n=12), as well as 1 participant who terminated CBT-TBI early but completed end-of-study assessments. Participants in this subsample of the ongoing RCT (n=18) were between the ages of 21 and 69 years (mean 39.2 years, SD 16.3 years), and the majority were white (n=14, 78%), non-Hispanic or Latino (n=15, 83%), and highly educated (n=10, 56% with at least 4 years of college). Just over half of the sample (n=10, 56%) were women, while less than half the sample (n=8, 44%) were married or in a relationship.

### Procedures

All study procedures, including pandemic-related modifications, were approved by the Massachusetts General Hospital Institutional Review Board. Adults with clinically significant depressive symptoms and a history of moderate-to-severe TBI were included in the study (see Multimedia Appendix 1 for the full study criteria [15-18]).

**Figure 1:**
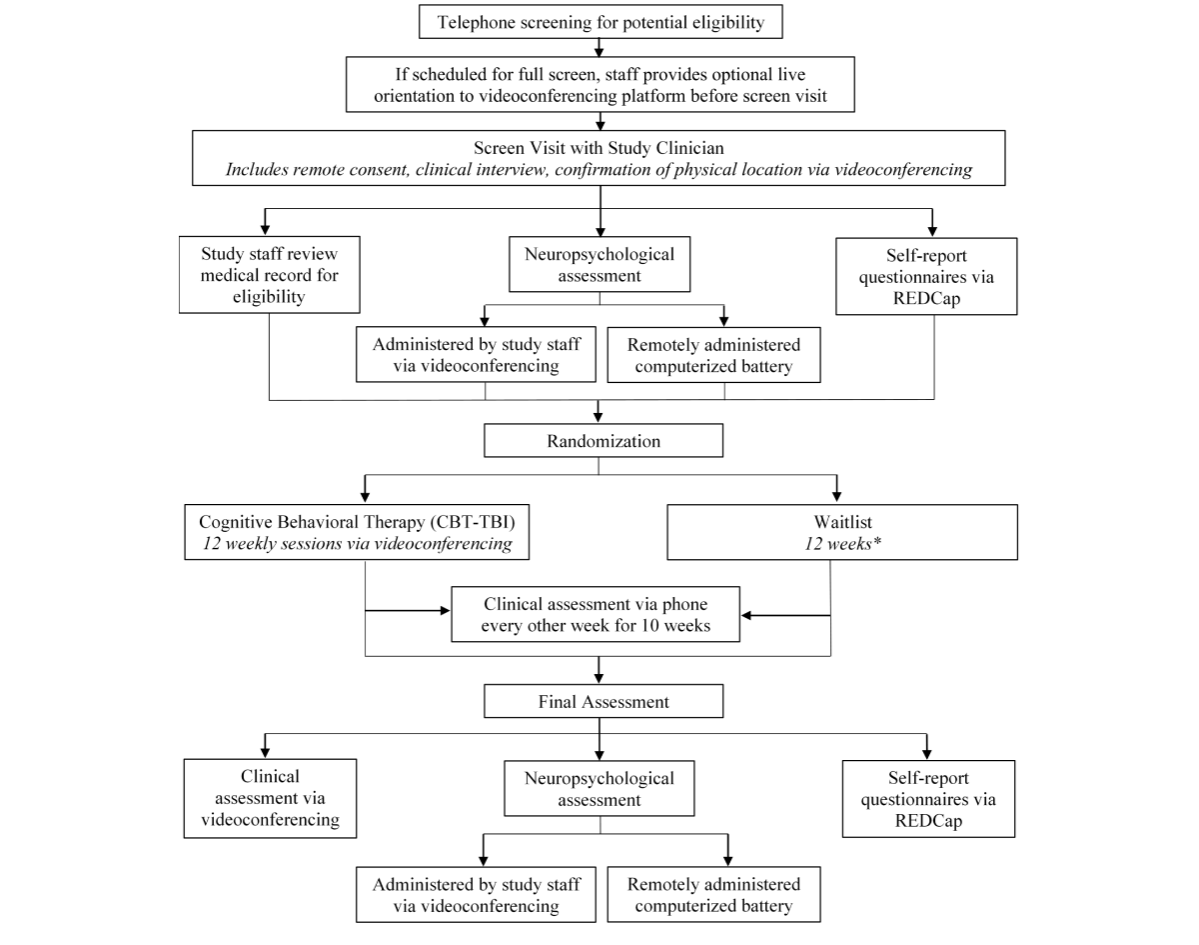
Flowchart of study procedures for eligible participants. *All participants randomized to waitlist can complete the intervention following the final assessment. CBT-TBI: cognitive behavioral therapy for depression in individuals with traumatic brain injury.

**Figure 2:**
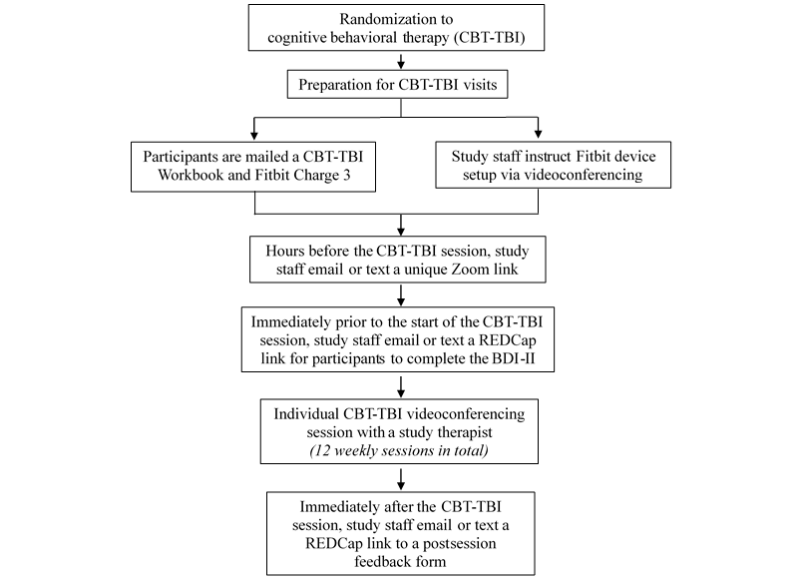
Flowchart of study procedures for treatment phase. BDI-II: Beck Depression Inventory-II; CBT-TBI: cognitive behavioral therapy for depression in individuals with traumatic brain injury.

The initial screening visit included informed consent, diagnostic and symptom evaluation with a study clinician, and a neuropsychological battery that was completed in-person prior to March 2020 and remotely since April 2020. Participants also completed a series of baseline self-report measures of mood; suicidality; and cognitive, social, and emotional functioning, using a secure web-based platform (REDCap). Eligible participants were then randomized to 12 weeks of a newly developed manualized cognitive behavioral treatment for depression adapted for individuals with TBI (CBT-TBI) or a 12-week waitlist. The intervention included psychoeducation, behavioral activation, goal setting, cognitive restructuring, and relapse prevention. CBT-TBI was adapted for individuals with TBI by incorporating the following strategies: repetition, patient workbook with session summaries and forced choice worksheets, modified thought records, therapeutic use of neuropsychological testing results, individually tailored text messages/between-session reminders, and daily use of an activity monitoring device (Fitbit Charge 3). Individuals in both conditions completed bimonthly phone assessments of depressive symptoms with an independent evaluator. Weekly 50 to 60-minute individual CBT-TBI sessions were delivered by a master’s or doctoral-level clinician in-person until March 16, 2020, and via Zoom videoconferencing (or telephone, when needed) thereafter. At the end of 12 weeks, all participants completed a postassessment, which included clinician-rated and self-rated measures, as well as repeat neuropsychological testing. Individuals randomized to the waitlist condition could receive CBT-TBI upon completion of 12 weeks of assessment. All study procedures are outlined in Figure 1, and a detailed breakdown of CBT-TBI visits is shown in Figure 2.

### Measures

#### Acceptability

The 12-item self-rated Satisfaction with Therapy and Therapist Scale-Revised (STTS-R) [19] was used to assess satisfaction in 2 domains of treatment. Current analyses included the satisfaction with therapy subscale scores, which range from 6 to 30, with higher scores indicating greater satisfaction. In March 2020, 5 questions were composed by the study team to gather feedback on remote CBT-TBI visits, including satisfaction with remote CBT-TBI sessions on a 5-point Likert scale (1, very satisfied to 5, very dissatisfied). Participants were also asked to share what they liked and did not like about virtual treatment. Participants who completed some CBT-TBI sessions in-person and some remotely were asked to indicate the degree of their preference for one modality over the other on a 5-point Likert scale (1, strongly preferred telemental health sessions to 5, strongly preferred in-person sessions). Finally, participants were asked to select the modality they would choose if given the option for treatment after the pandemic (eg, in-person, over the telephone, videoconferencing, and combination of in-person and virtual).

#### Safety

Suicidal ideation was monitored weekly during CBT-TBI with the suicide item from the Beck Depression Inventory-II (BDI-II) [20], a 21-item self-report scale designed to measure the presence and severity of depressive symptoms. The BDI-II suicide item is associated with the risk of repeat suicide attempts and death by suicide and is recommended as a screener for suicide risk in routine clinical care [21]. Adverse events were also assessed during bimonthly phone assessments.

### Study Modifications With Transition to Remote Implementation of Research

Several protocol modifications (Table 1) were instituted after all clinical trials for nonlifesaving interventions were halted in our institution due to the pandemic. Modifications aimed to facilitate feasibility and adherence to the original procedures as much as possible.

**Table 1:** Study modifications with transition to remote implementation.

Protocol element	In-person implementation	Remote implementation
Treatment modality	Individual face-to-face sessions in the office	Individual sessions via secure videoconferencing
Consent	Clinicians and participants review paper consent and sign in the office	Clinicians and participants utilize teleconsent with the REDCap eConsent template during videoconference
Data management	Participants complete questionnaires directly on REDCap using an in-office computer (preferred method) REDCap links are emailed to participants who are unable to complete questionnaires during office visit Paper copies are completed in office or at home for participants unable to complete electronically Clinicians complete pencil and paper assessments (requires data entry)	Via text or email, participants are sent a REDCap link to complete questionnaires independently (preferred method) Paper copies are mailed with self-addressed return envelope for participants unable to complete electronically Clinicians enter clinical data directly into REDCap
Neuropsychological assessment	Administered by study staff in the office (traditional measures): TOPF^a^ [22] WAIS-IV^b^ Coding, Digit Span and Similarities [23] D-KEFS^c^ Color Word and Trails [24] CVLT-II^d^ [25] Administered on an iPad in the office: NIH^e^ Toolbox Cognition Battery [26]	Administered by study staff via videoconferencing (traditional measures) TOPF [22] WAIS-IV Digit Span and Similarities WMS-IV^f^ Logical Memory I and II [27] Computerized battery completed by participants at home CNS Vital Signs [28]
Suicide risk monitoring	Clinicians review the paper copy responses to the BDI-II^g^ suicide item at the start of the CBT-TBI^h^ visit with participants in the room	The study coordinator reviews REDCap responses to the BDI-II suicide item at the start of the CBT-TBI visit and alerts clinicians to scores of 2 or higher
Preparation for CBT-TBI visits	Routine scheduling; the study coordinator answers questions from participants	A “Welcome Letter” is sent to establish expectations: Ensure security (eg, close other applications while Zoom is open) Ensure privacy (eg, conduct sessions in a private room with the door closed, use headphones and/or a noise blocker) Provides tips for limiting distractions (eg, silence cell phone, avoid eating, ensure device is fully charged, and let others in the home/space know you are unavailable) Consider the feasibility of your device (eg, a computer allows for typing notes in electronic handouts and hardwired ethernet connections can be more reliable than Wi-Fi)
Delivery and setup of wearable technology	The study coordinator sets up Fitbit with participants on the day of the first in-office CBT-TBI session	The study coordinator mails the device to participants and guides them through device setup via videoconferencing
CBT-TBI delivery modification	Provide handouts in the session that are added to the CBT-TBI Workbook every week	Mail the CBT-TBI Workbook with handouts and worksheets prior to the start of treatment Minimize reliance on screens (eg, turn away from the computer and turn off video) Tailor delivery to individual needs/preferences and be flexible (eg, utilize “screen share” and provide electronic handouts)

^a^TOPF: Test of Premorbid Functioning.

^b^WAIS-IV: Wechsler Adult Intelligence Scale–Fourth Edition.

^c^D-KEFS: Delis-Kaplan Executive Function System.

^d^CVLT-II: California Verbal Learning Test–Second Edition.

^e^NIH: National Institutes of Health.

^f^WMS-IV: Wechsler Memory Scale–Fourth Edition.

^g^BDI-II: Beck Depression Inventory-II.

^h^CBT-TBI: cognitive behavioral therapy for depression in individuals with traumatic brain injury.

#### Videoconferencing Platform

All procedures (except for neuropsychological testing, discussed below) that were previously conducted face-to-face were completed remotely using videoconferencing. Secure Zoom videoconferencing (Zoom Enterprise) was adopted after working with our institution’s Research Information Security Office to optimize privacy and security settings, including enabling the waiting room, locking meetings once sessions begin, and generating meeting IDs with a password. Individuals who were unfamiliar with the videoconferencing platform received step-by-step instructions for Zoom account set-up, and could participate in a “trial run” and orientation to the platform with the study coordinator.

#### Remote Consent

Since the study transitioned to remote implementation, the informed consent process was embedded into a live telehealth session, also referred to as teleconsent [29]. An institution-specific REDCap electronic informed consent template was utilized. The study clinician met with the participant over Zoom to review the consent form and instruct the participant to digitally sign consent. A signed copy was then securely emailed directly to the participant from REDCap. Participants were given the option to receive a mailed paper copy and/or a brief summary of key study information to make the process less overwhelming. Overall, teleconsent provides a feasible alternative to in-person paper consent and facilitates research continuity when face-to-face interactions are not possible [30].

#### Data Management

Prior to the pandemic, study participants were given the option of completing self-report questionnaires directly in REDCap using a computer or tablet, either in our office or at home. Individuals participating remotely were provided an electronic link to complete questionnaires at home directly on REDCap. Given that it can be cognitively taxing to sit at a screen for an extended duration of time, participants were encouraged to complete a few questionnaires at a time. Participants were offered the option of being mailed paper questionnaires with a self-addressed envelope that was returned to the research team.

#### Neuropsychological Testing

The original in-person battery included a series of traditional paper and pencil neuropsychological measures and the iPad-administered NIH Toolbox Cognition battery [26]. Following a review of the available teleneuropsychology literature [31] and guidelines for remote assessments [32], it was determined that certain subtests from the original battery could be administered via videoconferencing, likely without significant impact on reliability and validity, although some tests could not (Table 1). CNS Vital Signs [28], a brief computerized neurocognitive test battery, replaced the NIH Toolbox Cognition battery and was administered remotely in accordance with guidelines to maximize validity. Participants were instructed to watch a preparatory video that emphasizes the importance of creating an optimal standardized testing environment (eg, limit distractions and interruptions, and set aside sufficient time to complete), which in turn maximizes the reliability of test results. CNS Vital Signs reports include a validity indicator for each subtest, allowing the clinician to follow-up with the participant about possibly invalid results.

#### Suicide Risk Assessment

On the day of CBT-TBI sessions, the study coordinator emailed or texted the REDCap link for the BDI-II [20] to the participants, instructed them to complete the measure prior to meeting with the study therapist, and reviewed their responses to the BDI-II suicide item in real-time. If the participant did not complete the measure, the study therapist was notified to remind the participant to do so and to review the suicide item response before starting the session. In the event that the participant had a score of 2 or higher, the therapist was immediately alerted to conduct a detailed suicide risk assessment in the session and to determine the need for a higher level of care, which could involve voluntary or involuntary hospitalization and/or contacting the individual’s previously identified emergency contact. The participant’s physical location was identified after signing consent and was confirmed before starting every CBT-TBI session. All efforts were made for the study therapist to remain connected to the participant (on Zoom or telephone) until emergency personnel arrived at their location.

#### Preparing for CBT-TBI Telehealth Visits

Given that individuals with TBI can be sensitive to changes in routine due to deficits in mental flexibility and problem solving [33], it is encouraged that telemental health visits mirror in-person CBT-TBI visits as much as possible. A predictable environment that parallels the in-person setting (ie, consistent office space/background) may be beneficial [34]. In order to compensate for a patient’s reduced ability to read the therapist’s nonverbal cues over video, clinicians configured their camera to ensure that the patient could see as much of their body language as was feasible [34]. Clear and consistent expectations about virtual visits were directly communicated in a “Welcome Letter” that emphasized how best to ensure security and privacy, and provided tips for limiting distractions during the session (Table 1). Participants who had more than one internet and video-enabled device were encouraged to consider which device would best suit their needs based on factors such as strength of internet connection and device portability. Additionally, the active hands-on nature of the study intervention, which uses worksheets and encourages notetaking, warranted an appropriate workspace, such as sitting at a desk or table.

#### Delivery and Setup of Wearable Technology

Participants were mailed a Fitbit Charge 3 activity tracker prior to starting the intervention. The study coordinator scheduled individual videoconferencing meetings with participants prior to the first CBT-TBI session where they provided instruction on setup and use, and provided a handout reiterating this information.

#### Minimize Reliance on Screens

For some individuals with TBI, screen time can exacerbate symptoms, including headaches due to photosensitivity [35]. To reduce the degree to which participants engaged with material in electronic format, all participants were mailed a physical copy of the CBT-TBI Workbook, which contained copies of weekly agendas, handouts, and worksheets. Participants who reported considerable difficulty with photosensitivity were encouraged to turn away from the computer or turn off video.

#### Importance of Tailoring Delivery and Being Flexible

Given the importance of clear direct communication for individuals experiencing cognitive difficulties, the therapist spoke with the participants in the first session to understand their comfort with technology and preferences for ways of engaging in the collaborative treatment (eg, physical vs electronic worksheets, therapist vs participant typing responses into worksheets, and use of the videoconferencing screen share feature to provide visual cues and allow the therapist to model skill use). Consistent with procedures utilized during in-person delivery of the intervention, participants continued to receive between-session reminders via text, email, or phone call to carry out collaboratively identified activities or goals (eg, behavioral activation).

#### Troubleshooting Challenges With Technology

It is inevitable that technological challenges will arise both prior to and during virtual sessions. It is important that clinicians do not get visibly frustrated in the face of technological difficulties, as the patient may interpret this as the clinician being upset with them [34]. Additionally, shared insecurities over technology may, in fact, aid therapeutic alliance [36]. However, it is worth recognizing that technological issues can be disruptive, and clinicians may want to identify a cutoff point at which they switch from videoconferencing to a telephone session. In the first CBT-TBI session, the clinician and participant develop an individualized plan for navigating potential technological difficulties, such as losing a connection mid-session.

### Data Analyses

To assess the preliminary feasibility and acceptability of remote study implementation, descriptive statistics were used to report the number of CBT-TBI intervention sessions attended, number of assessment sessions attended, number of participants who completed the study, and rate of satisfaction with treatment (STTS-R and supplemental questions). Study retention (number of study completers/number randomized) was also calculated. Finally, the number of elevated responses to the BDI-II suicide item (≥2) was described to demonstrate the maintenance of safety protocols.

## Results

### Feasibility

At the time of the transition to remote procedures (March 2020), there were 9 active study participants, including 3 participants on the waitlist (ie, had not started CBT-TBI) and 6 participants who were mid-treatment. The 6 participants who started CBT-TBI in-person prior to March 2020 were at different points in treatment at the time of the transition (weeks 3, 7, 9, 10 11, and 12), and all went on to complete the remainder of their 12 weeks of CBT-TBI remotely using telemental health sessions. Two out of three waitlist participants who enrolled in the study before March 2020 with the expectation of attending CBT-TBI sessions in-person completed the entire 12 weeks of CBT-TBI via videoconferencing. One participant discontinued participation after 4 CBT-TBI sessions due to a demanding work schedule but completed postassessments. Finally, of all randomized participants who enrolled in the study remotely (n=9), 7 (78%) completed the study, 1 (11%) was withdrawn due to worsening depression, and 1 (11%) remains active in CBT-TBI. The study retention rate prior to March 2020 was 100% (8 CBT-TBI completers), and completion from March through January 2021 was about 93% (13 out of 14 possible randomized CBT-TBI completers). Approximately 91% of clinician-rated assessments (102 out of 112 possible assessments) were completed throughout the period in which the study had been conducted remotely.

### Acceptability

Satisfaction with therapy (STTS-R therapy subscale) was high (mean 27.1, SD 2.8) among participants who completed at least one session of CBT-TBI remotely (n=16). Overall satisfaction with telemental health sessions (videoconferencing, telephone, or a combination of both) was high (n=14); 9 participants (64%) reported being “very satisfied,” 4 participants (29%) were “satisfied,” and 1 participant (7%) reported being “neither satisfied nor dissatisfied.” If given a choice of modality in the future (n=14), 3 participants (21%) indicated that they would choose in-person treatment, 4 participants (29%) indicated that they would choose telemental health treatment (videoconferencing or telephone), and 7 participants (50%) indicated that they would choose a combination of in-person and telemental health treatment. Among the participants who completed a combination of in-person and telemental health treatment and provided feedback (n=4), there was no clear pattern in preferred modality, as they reported strongly preferring telemental health treatment (n=1, 25%), somewhat preferring telemental health treatment (n=1, 25%), strongly preferring in-person treatment (n=1, 25%), and no indication of preference (n=1, 25%).

Qualitative feedback highlighted that all 14 study completers noted at least one benefit of telemental health sessions, including ease of conducting sessions from home and not having to travel for appointments. Conversely, technological challenges, reduced focus, limited privacy, and difficulty feeling connected with the therapist were noted as factors that participants disliked about telemental health sessions. Five participants reported that there was nothing they disliked about telemental health treatment.

### Safety

Since March 2020, 1 participant had a score of 3 on the BDI-II [20] suicide item in 2 consecutive weeks. Per protocol, the study coordinator alerted the CBT-TBI therapist and the principal investigator immediately after having identified the safety concern, and the therapist started the CBT-TBI session with a thorough assessment of suicide risk. After several weeks of worsening depression and increasing suicidal ideation, the participant was eventually referred for a higher level of care (partial hospitalization program), was withdrawn from the study, and was ultimately hospitalized voluntarily for worsening of symptoms. No serious adverse events were reported throughout the duration of remote procedures.

## Discussion

### Principal Findings

Using several procedural modifications described in this paper, an in-person RCT of CBT for depression after TBI was converted to remote implementation and demonstrated preliminary evidence of feasibility, acceptability, and safety. Specific modifications to study implementation and the treatment protocol are outlined in Table 1. Given the range of symptoms and deficits that can arise after TBI (eg, photosensitivity and impaired attentional capacity), all modifications were made with consideration of their potential impact on participants and to enhance feasibility.

Preliminary data supported the feasibility, acceptability, and safety of conducting an RCT for depression among individuals with TBI exclusively utilizing remote procedures. Specifically, preliminary results demonstrated a high rate of completion for clinician-rated assessments (102/112, 91%) and high study retention (13/14, 93%). Procedures that were designed to monitor safety were effective in identifying individuals at high risk for suicide, triggering clinician suicide risk assessments via videoconferencing. Feedback from participants suggested a high degree of satisfaction with the CBT-TBI treatment and telemental health modality, providing initial evidence of the acceptability of the remotely delivered study intervention. The findings are consistent with the results of previous studies that have examined telephone-delivered cognitive behavioral interventions among individuals with TBI [11,37].

Feedback from our small sample highlighted a range of preferences when participants were asked to consider their ideal treatment modality (in-person treatment, telemental health treatment, or a hybrid model), which has significant implications for study participation and potentially for treatment outcomes. Research has demonstrated better treatment outcomes among individuals whose preferences about psychological treatment (eg, appointment time, venue, and treatment type) are accommodated compared with individuals whose preferences are not met [38]. Previous research among depressed individuals with TBI utilized choice-stratified randomization, in which participants could assert a preference for CBT that was delivered in-person or over the telephone prior to randomization, in order to enhance ecological validity [11]. Qualitative feedback from our study suggested that participants may appreciate a mix of in-person and telemental health visits, which is consistent with evidence for the high feasibility and acceptability of “blended” models of delivery (combination of face-to-face and web-based sessions) of CBT for depression [39]. Although the efficacy of our study intervention is unknown at this time, tailoring the intervention modality according to preferences may lead to greater attendance at treatment sessions and engagement in treatment.

### Limitations

It is important to acknowledge several limitations. Although several steps were taken to optimize the testing environment, neuropsychological assessment is ideally suited to in-person administration. Challenges with technology and suboptimal conditions in the participant’s environment have the potential to impact engagement and data collection. Behavioral observations can be restricted by videoconferencing, and rapport can sometimes be limited without in-person interactions, which may impact participant responses or commitment to participation, especially prior to randomization. It is also important to note that our sample was heavily comprised of individuals who received specialized acute inpatient rehabilitation (n=11, 61%) and specialized outpatient treatment (n=13, 72%) for their TBIs in a single academic medical center in the Northeast. Individuals who receive inpatient rehabilitation represent 7% of all persons hospitalized with moderate-to-severe TBI, are less likely to be a member of a racial/ethnic minority group, and are more likely to have health insurance compared with individuals who are hospitalized and do not receive inpatient rehabilitation after moderate-to-severe TBI [40]. Thus, our sample may not be representative of all individuals with moderate-to-severe TBI in the United States.

### Conclusion

Remote study participation has been a feasible alternative when in-person research was halted during the COVID-19 pandemic. Strategic procedural modifications outlined in this paper have been instrumental to the continued feasibility of recruitment and retainment of individuals with depression and TBI in the context of our ongoing RCT. Furthermore, telemental health offers significant advantages in eliminating common barriers to study participation, including transportation, time needed to travel to appointments, distance to the hospital, limited mobility, and inclement weather. Conversely, some individuals may struggle to secure private space for their sessions, and a visit to a traditional office space may be preferred. Further, many individuals do not have internet access and a camera-enabled device for videoconferencing. Some individuals may find it easier and less intimidating to be vulnerable about the challenges they face over a computer screen rather than in-person [41], while others may have difficulty connecting with a therapist through a screen. For some individuals, the flexibility of utilizing both types of modalities within the course of treatment may be an ideal balance; thus, future research designs should consider the role of patient preference. For individuals with TBI who frequently struggle with physical, cognitive, and emotional impairments, flexibly tailored treatments that utilize telemental health and in-person modalities are likely to be important in both research and clinical settings. Future research should directly compare the feasibility and efficacy of CBT delivered via telemental health, in-person, and hybrid models for individuals with TBI, as well as the validity and reliability of remote neuropsychological assessments and strategies to facilitate remote engagement. The COVID-19 pandemic abruptly presented researchers with unique challenges that have required flexibility and innovation. The advantages presented by the ability to conduct clinical research using remote methods are likely to persist long after the pandemic ends.

## Appendix

Multimedia Appendix 1 Inclusion/exclusion criteria for the randomized controlled trial.

Multimedia Appendix 2 CONSORT checklist.

## Abbreviations

BDI-II: Beck Depression Inventory-II
CBT: cognitive behavioral therapy
CBT-TBI: cognitive behavioral therapy for depression in individuals with traumatic brain injury
MDD: major depressive disorder
NIH: National Institutes of Health
RCT: randomized controlled trial
STTS-R: Satisfaction with Therapy and Therapist Scale-Revised
TBI: traumatic brain injury
