# Supplementary material for: Transitioning From In-Person to Remote Clinical Research on Depression and Traumatic Brain Injury During the COVID-19 Pandemic: Study Modifications and Preliminary Feasibility From a Randomized Controlled Pilot Study
Source: JMIR Form Res. 2021 Dec 1;5(12):e28734. doi: 10.2196/28734 (PMC8638786; doi:10.2196/28734)
Supplement: Multimedia Appendix 1 [file formative_v5i12e28734_app1.docx]

**Multimedia Appendix 1.** Inclusion/exclusion criteria for the randomized controlled trial.

Eligibility criteria include: 1) age 18 or older; 2) having been treated for complicated mild to severe TBI that occurred at least 3 months prior to study entry; 3) clinically significant depressive symptoms [meets criteria for Major Depressive Episode on the MINI [15] or has a total score ≥ 23 on the clinician-rated Inventory of Depressive Symptomatology (IDS-C)] [16]; 4) out of post-traumatic amnesia (PTA) at time of enrollment; 5) English language proficiency; 6) ability to attend in-person, outpatient sessions (April 2019-March 2020) or access a smartphone/tablet/computer with internet and video capabilities for virtual sessions (April 2020-present); 7) ability to see and hear (hearing or visual loss cannot impair ADLs or in-room conversation); and 8) possesses capacity for consent (University of California San Diego Brief Assessment of Capacity to Consent [17]). TBI severity criteria were defined such that participants have to meet one of the following criteria in order to qualify for the study: a) Glasgow Coma Score (GCS) [18] 3–12 with GCS motor score ≤ 5 within 4 hours after injury; b) GCS 3–12 with GCS motor score = 6 within 4 hours after injury AND documented intracranial abnormalities on imaging; c) GCS 13–15 within 4 hours after injury AND documented intracranial abnormalities on imaging; d) loss of consciousness (LOC) > 30 min; or e) post-traumatic amnesia (PTA) > 24 hours. Participants are not included in the study if any of the following exclusionary criteria are met: 1) presence of uncontrolled medical illness; 2) presence of behavioral dyscontrol; 3) evidence of acute PTSD as the primary diagnosis; 4) evidence of substance use disorder, moderate or severe, within the past six months; 5) history of bipolar disorder, a primary psychotic disorder or current psychotic symptoms, or acute suicidality or homicidality; 5) currently receiving regular (≥ 2 times/mo.) psychosocial treatment for depression; 6) participated in CBT for depression within the past 6 months, and 7) history of dementia or severe cognitive impairment that is not related to TBI.
